# Soil microbial diversity–biomass relationships are driven by soil carbon content across global biomes

**DOI:** 10.1038/s41396-021-00906-0

**Published:** 2021-02-09

**Authors:** Felipe Bastida, David J. Eldridge, Carlos García, G. Kenny Png, Richard D. Bardgett, Manuel Delgado-Baquerizo

**Affiliations:** 1 CEBAS-CSIC. Department of Soil and Water Conservation, Campus Universitario de Espinardo, 30100 Murcia, Spain; 2 Centre for Ecosystem Studies, School of Biological, Earth and Environmental Sciences, University of New South Wales, Sydney, NSW 2052 Australia; 3 Department of Earth and Environmental Sciences, Michael Smith Building, The University of Manchester, Oxford Road, Manchester, M13 9PT UK; 4 Asian School of the Environment, Nanyang Technological University, 50 Nanyang avenue, Singapore, Singapore 639798; 5 Departamento de Sistemas Físicos, Químicos y Naturales, Universidad Pablo de Olavide, 41013 Sevilla, Spain

**Keywords:** Biodiversity, Microbial ecology

## Abstract

The relationship between biodiversity and biomass has been a long standing debate in ecology. Soil biodiversity and biomass are essential drivers of ecosystem functions. However, unlike plant communities, little is known about how the diversity and biomass of soil microbial communities are interlinked across globally distributed biomes, and how variations in this relationship influence ecosystem function. To fill this knowledge gap, we conducted a field survey across global biomes, with contrasting vegetation and climate types. We show that soil carbon (C) content is associated to the microbial diversity–biomass relationship and ratio in soils across global biomes. This ratio provides an integrative index to identify those locations on Earth wherein diversity is much higher compared with biomass and vice versa. The soil microbial diversity-to-biomass ratio peaks in arid environments with low C content, and is very low in C-rich cold environments. Our study further advances that the reductions in soil C content associated with land use intensification and climate change could cause dramatic shifts in the microbial diversity-biomass ratio, with potential consequences for broad soil processes.

## Introduction

In ecology, the relationship between biodiversity and biomass has been a long standing debate which originated in plant communities studies by postulating that resource availability is a key regulator of plant productivity and/or biomass [1], and that there is a unimodal (or ‘humped-back’) relationship between plant diversity and productivity [2–5]. This humped-back diversity–biomass relationship is often attributed to multiple complementary processes, ranging from resource stress (under low plant biomass levels) where biomass and diversity are often positively associated, to competitive exclusion (under high biomass levels) where a few species dominate the resources and biomass is negatively correlated with diversity [6–11]. The evidence for this humped-back model in plant communities has extended in the last years [3, 12], while other studies have found no clear relationships between productivity and richness [13].

The diversity and biomass of soil microbial communities are the major regulators of fundamental ecosystem processes, such as organic matter decomposition, nutrient cycling, and gaseous fluxes [14–16]. However, although our understanding of biotic and abiotic factors controlling soil microbial diversity and biomass is increasingly growing [17–19], remarkably little is known about how soil microbial diversity and biomass are related across global biomes, and the factors that control such relationships [20, 21]. Moreover, mechanistic modeling, as such provided by Grace et al. [3] in plant communities, needs to be applied in order to properly understand the relationships between diversity and biomass in soil microbial communities. This information is critical if we are to understand how microbial-driven processes are regulated in a changing planet. Some studies have argued that competitive exclusion is more important for aboveground than for belowground communities, mainly because many organisms are spatially separated in soil [20, 21]. However, more recent studies are challenging this view by providing evidence of competitive exclusion associations between soil microbial communities in polar soil ecosystems [22] and elsewhere [23]. In soil, flows of carbon (C) fuel belowground productivity and microbial biomass [15, 24]. Moreover, recent within-biome field studies and microcosm experiments have revealed a strong correlation between soil organic C content and microbial diversity [17, 22, 25, 26]. Thus, since soil C is highly vulnerable to global change drivers such climate and land use intensification [27, 28], changes in its content might result in important imbalances in the microbial diversity-to-biomass relationship.

Herein, we hypothesize that soil C content is an important driver of the relationship between soil microbial diversity and biomass across global biomes [22] and that the microbial diversity-to-biomass ratio is an integrative proxy to know how diversity and biomass are interlinked. Two conceptual alternatives would support these expectations. First, the Stress Gradient Hypothesis suggests that positive species interactions such facilitation and specialization are more important in stressful environments (such those soils under more arid conditions and with low soil C content) than in more benign ones where competition should be more common [29, 30]. Thus, we expect relatively higher diversity in comparison to biomass (higher diversity-to-biomass ratios) in soils with low soil C content such those located in dry forests, shrublands, and cold forests. Second, in environments with higher soil C content, an increase in microbial biomass is potentially associated with the ecological displacement of non-competitive populations and a reduction of diversity (competitive exclusion; *sensu* Grace [6], Grime [7]), as proposed in plant communities [2, 4, 5, 9, 10] and local soil microbial communities [22]. Thus, we expect humped-back relationships and reductions of the diversity-to-biomass ratio in soil C-rich environments. Further, microbial biomass and diversity are, in theory, needed to support soil processes [15, 31–34]. However, we also know that some generalized processes (conducted by multiple soil organisms), such as soil respiration (organic matter mineralization) are comparatively more expected to be influenced by microbial biomass than by diversity, as the microbial machinery required for decomposition is widely phylogenetically shared across soil taxa. In this respect, for broad processes, such as organic matter mineralization, a greater microbial biomass (i.e., in soils with high C content) will have comparatively major effect on soil respiration than diversity because biomass is usually correlated to respiration [34, 35]. Following this theoretical framework, we expect that increases in microbial diversity-to-biomass ratio negatively influence soil organic matter mineralization across global biomes.

To fill this gap of knowledge, we conducted a cross-biome field survey of 435 soil samples taken from 87 locations across five continents, thereby encompassing a wide range of ecosystem and climate types. Our goal was to examine relationships between microbial diversity and biomass in soil and their consequences for ecosystem functions, and to identify the dominant environmental factors that control these relationships across biomes. Our survey included information on bacterial and fungal diversity (from amplicon sequencing methods) and biomass (i.e., phospholipid fatty acids, PLFA), combined with environmental data associated with multiple soil abiotic properties, climatic properties, and vegetation attributes. We focused on bacteria and fungi because they constitute the most diverse and abundant microbial communities on Earth.

## Material and methods

### Field survey and soil sampling

Field data were collected between 2016 and 2017 from 87 locations across nine countries and five continents (Supporting Information Fig. S1). These locations include a wide range of soil, vegetation (including cold forests, dry forests, forblands, grasslands, moss heaths, shrublands, temperate forests, tropical forests, and croplands), and climate (tropical, temperate, continental, polar, and arid) types. Sampling was designed to obtain wide gradients of edaphic characteristics. Field surveys were conducted according to a standardized sampling protocol [36]. In each location, we surveyed a 50 × 50 m plot. Five composite soil samples (five soil cores/sample; 0–10 cm depth) were randomly collected within 50 × 50 m plots of each location under the various dominant plant species of that ecosystem type for a total of 435 samples in this study [37]. Our approach was explicitly designed to account for within-plot heterogeneity in microbial diversity and biomass by including five soil composite samples within each of the globally distributed 87 plots. Plant material was removed from soil samples before sieving. Three parallel transects of 30 m, spaced 25 m apart, were added. The cover of perennial vegetation was measured in each transect using the line-intercept method [36]. Plant cover ranged between 0 and 100%. Following field sampling, soils were sieved (<2 mm) and frozen at −20 °C for microbial analyses. Other soil fraction was air-dried for chemical analyses.

### Soil chemical and physical analyses

For all soil samples (*n* = 435), we measured electrical conductivity, pH, texture (clay plus silt content), available P (Olsen P), and soil organic C (SOC) content as % (soil C hereafter). Soil properties were determined using standardized protocols described elsewhere [36]. Soil pH was measured in every soil sample with a pH meter, in a 1: 2.5 mass: volume soil and water suspension. Soil texture (% of fine fractions: clay + silt) was determined according to Kettler et al. [38]. Total *N* was obtained using a CN analyzer (LECO CHN628 Series, LECO Corporation, St Joseph, MI USA). SOC content ranged between 0.1% and 38%, available P between 0.5 and 72 mg P kg^−1^ soil, pH between 3.8 and 9.1, and the % of clay + silt varied between 0.3% and 86%, respectively.

### Microbial biomass and respiration

Soil microbial biomass of each soil sample (*n* = 435) was estimated from PLFAs extracted from a 0.5 g freeze-dried subsample, by using the method described in Bligh and Dyer [39] and modified by Buyer and Sasser [40]. The extracted fatty acid methyl esters were analyzed on an Agilent Technologies 7890B gas chromatograph with an Agilent DB-5 ms column (Agilent Technologies, CA, USA). The fatty acids selected to represent bacterial biomass are the PLFAs i15:0, a15:0, 15:0, i16:0, 16:1ω7, 17:0, i17:0, a17:0, cy17:0, 18:1ω7, and cy19:0, and the fatty acid representative of fungal biomass is the 18:2ω6 [41, 42]. Soil microbial respiration rates were determined on a composite soil samples per plot (*n* = 87) by quantifying the CO_2_ released during 16 days from 1 g of soil sample incubated at 28 °C and 50% of water holding capacity in 20-ml glass vials in the dark, after a 1-week pre-incubation [14]. We are aware that only one fatty acid (18:2ω6) is usually selected as indicator of fungal biomass, while it can be also originated from other eukaryotic cells (i.e., plants). However, several studies have highlighted that this fatty acid is often well correlated with the fungal marker ergosterol [43, 44].

### Soil microbial diversity

The diversity of soil bacteria and fungi was analyzed through amplicon sequencing (Illumina MiSeq). A total of 10 g of frozen soil samples were cooled using liquid nitrogen and ground using a mortar and pestle. Soil DNA (*n* = 435) was extracted using a DNA Isolation Kit (Powersoil, MoBio Laboratories, Carlsbad, CA, USA). A portion of the bacterial 16S and eukaryotic 18S rRNA genes were sequenced using the 515F/806R and Euk1391f/EukBr primer sets [45, 46], respectively. Analyses of bioinformatics were carried out with QIIME [47], USEARCH [48], and UNOISE3 [48]. Phylotypes (i.e., Amplicon Sequencing Variants, ASVs) were identified at the 100% identity level. The ASV abundance tables were rarefied at 5000 (bacteria via 16S rRNA gene) and 2000 (fungi via 18S rRNA gene), respectively to ensure even sampling depth. The diversity (richness) of soil bacteria and fungi was determined from rarefied ASV abundance tables. Before conducting statistical modeling, we also ensured that our choice of rarefaction level, taken to maximize the number of samples in our study, was not obscuring our results [37]. Rarefaction curves are showed in Fig. S2. Before conducting statistical modeling, we ensured that our choice of rarefaction level, taken to maximize the number of samples in our study, was not obscuring our results. Thus, using the samples with the highest sequence/sample yield, we tested for the impact of different levels of rarefaction on belowground diversity. Importantly, we found highly statistically significant correlations between the diversities and community compositions of soil bacteria (rarefied at 5000 vs. 18,000 sequences/sample) and fungi (rarefied at 2000 vs. 10,000 sequences/sample), providing evidence that our choice of rarefaction level did not affect our results or conclusions (*r* > 0.98; *p* < 0.001 in all cases). Microbial diversity data, but not PLFA results, were utilized in an earlier study aiming to study the evolution of soil microbial communities in global chronosequences [37].

### Standardized microbial richness-to-biomass ratio

We also calculated the richness-to-biomass ratio for bacterial and fungal communities. To do this, we first standardized the diversity and biomass of soil microbial communities between 0 and 1 to equally weight diversity and biomass before calculating their ratio. This ratio aims to provide a straightforward index to highlight those locations on Earth wherein diversity is much higher compared with biomass and vice versa.

### Statistical analyses

We tested for significant differences in microbial biomass and richness across major ecosystem types using one-way non-parametric permutational analysis of variance (PERMANOVA) and ANOVA. We then used the ‘rfPermute’ package in R to conduct Random Forest Analyses [49], as described in Delgado-Baquerizo et al. [50] to identify predictors of microbial richness in the global dataset. Further, we used linear or quadratic relationships to evaluate the direction and shape of the relationship between microbial biomass and microbial richness (independently for bacteria and fungi) as detailed elsewhere [50]. The best model fit was selected by identifying the model with the lowest Akaike information criteria index [51].

Further, we used structural equation modeling (SEM) [52] to evaluate the direct and indirect relationships among abiotic (pH, soil C content, and texture; clay+silt), biotic (microbial biomass, dominant vegetation types |forest and grasslands| and plant cover) and climatic (MAT and MAP) environmental factors on microbial richness effect based on expectations of an a priori model (Fig. S3; Table S1). Moreover, additional SEMs were also performed for the richness-to-biomass ratio. Evaluations of SEMs were carried out separately for bacteria and fungi. After attaining a satisfactory model fit, we introduced composite variables into our model. The use of composite variables does not alter the underlying SEM model, but collapses the effects of multiple conceptually related variables into a single composite effect, aiding interpretation of model results. Since some of the variables introduced were not normally distributed, the probability that a path coefficient differs from zero was tested using bootstrap tests. However, in such cases, bootstrapping tests do not assume that the data match a particular theoretical distribution.

### Mapping the distribution of microbial richness-to-bacterial ratio

We used the prediction-oriented regression model Cubist [53] to predict the distribution of microbial biomass and richness-to-biomass ratios across the globe as done in Delgado-Baquerizo [54]. The Cubist algorithm uses a regression tree analysis to generate a set of hierarchical rules using information on environmental covariates, based on real data (435 soil samples), which are later used for spatial prediction [55]. Our model includes information on soil carbon, major ecosystem types (forests and grasslands), soil pH and texture (% of clay + silt), and climate (MAT and MAP). The inclusion of these variables in our models was limited to the existence of high-resolution global maps. Information for other environmental predictors was not available at the global scale or was not comparable with our data. Global predictions are done on a 25 km resolution grid including 225530 locations. Global information on these predictors was obtained from global databases available online. Global information on soil properties for this grid was obtained using the ISRIC (global gridded soil information) SoilGrids (https://soilgrids.org/#!/?layer=geonode:taxnwrb_250m). Global information on climate was obtained from the WorldClim database (www.worldclim.org). Global information on the major vegetation types in this study (grasslands and forests) was obtained using the Globcover2009 map from the European Space Agency (http://due.esrin.esa.int/page_globcover.php). The R package Cubist was used to conduct these analyses [55]. The lack of alternative global databases including both diversity and biomass simultaneously limited our capacity to independently cross-validate our global maps. Future research will need to further evaluate and validate our mapping effort.

## Results and discussion

**Fig. 1:**
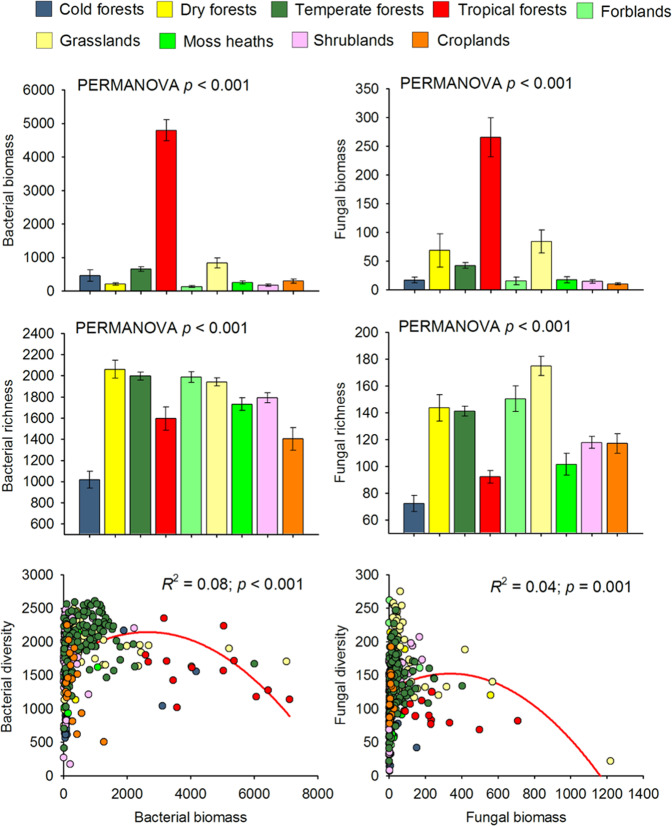
Microbial biomass (nmol PLFA g^−1^ dry soil) and richness in soil, and their relationships across globally distributed ecosystems.

**Fig. 2:**
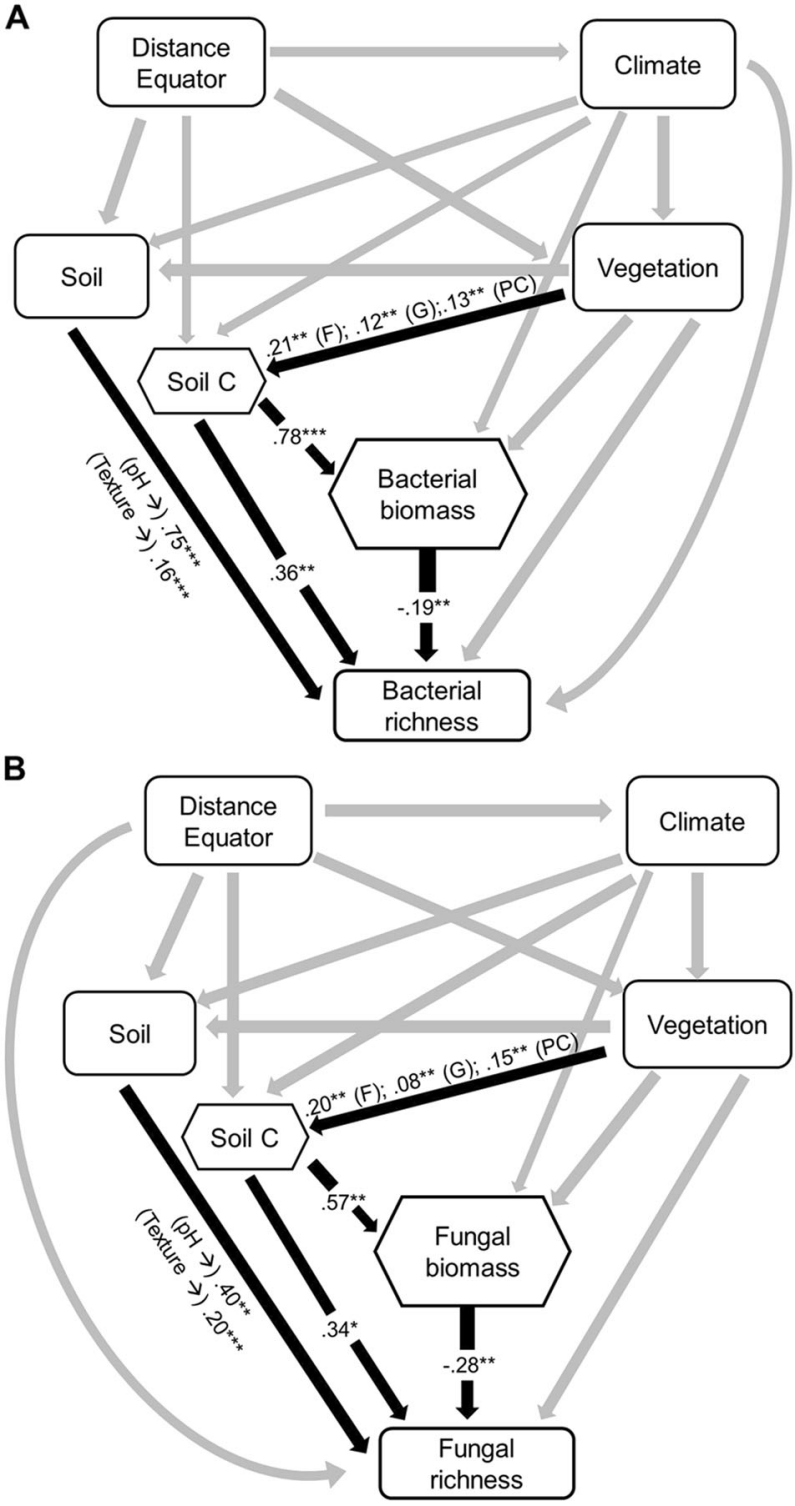
Structural equation models (SEMs) describing the effects of multiple predictors on microbial diversity. **A** Refers to bacterial communities and **B** Refers to fungal communities. Numbers adjacent to arrows and in boxes are indicative of the effect size (*p ≤ 0.05; **p ≤ 0.01; ***p ≤ 0.001) of the relationship. *R*^2^ denotes the proportion of variance explained. Climate includes mean annual precipitation (MAP) and mean annual temperature (MAT). Soil includes pH and texture. Vegetation includes plant cover (PC), grassland (G), and forest (F). Hexagons represent quadratic variables. The relationship between pH and bacterial richness was quadratic. There was a nonsignificant deviation of the data from the model for bacterial (*χ*^2^ = 0.28, df = 1; *p* = 0.60; RMSEA *p* = 0.74) and fungal (*χ*^2^ = 0.09, df = 1; *p* = 0.76; RMSEA *p* = 0.85) diversity. *R*^2^ as follows: Bacterial richness = 0.48; Bacterial biomass = 0.67; Fungal richness = 0.45; Fungal biomass = 0.39. Direct effects for bacterial and fungal SEM are provided in Supporting Information (Tables S3 and S4, respectively).

**Fig. 3:**
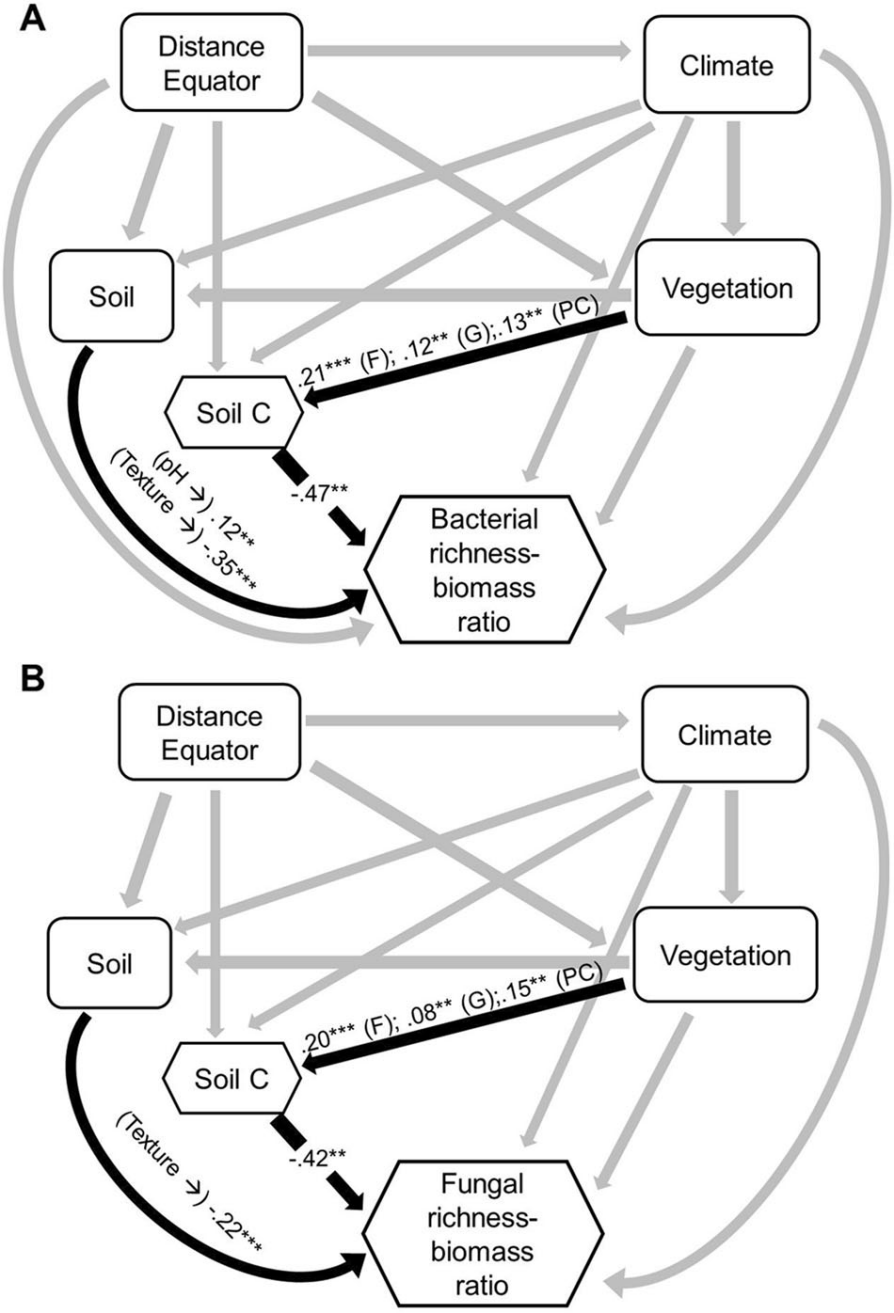
Structural equation models (SEMs) describing the effects of multiple predictors on microbial richness-to-biomass ratio. **A** Refers to bacterial communities and **B** Refers to fungal communities. Numbers adjacent to arrows and in boxes are indicative of the effect size (*p ≤ 0.05; **p ≤ 0.01; ***p ≤ 0.001) of the relationship. *R*^2^ denotes the proportion of variance explained. Climate includes mean annual precipitation (MAP) and mean annual temperature (MAT). Soil includes pH and texture. Vegetation includes plant cover (PC), grassland (G), and forest (F). Hexagons represent quadratic variables. The relationship between pH and bacterial richness was quadratic. There was a nonsignificant deviation of the data from the model for bacterial (*χ*^2^ = 0.38, df = 1; *p* = 0.54; RMSEA *p* = 0.70) and fungal (*χ*^2^ = 0.16, df = 1; *p* = 0.69; RMSEA *p* = 0.80) ratio. *R*^2^ as follows: Bacterial ratio = 0.68; Fungal ratio = 0.52. Direct effects for bacterial and fungal SEM are provided in Supporting Information (Tables S5 and S6, respectively).

**Fig. 4:**
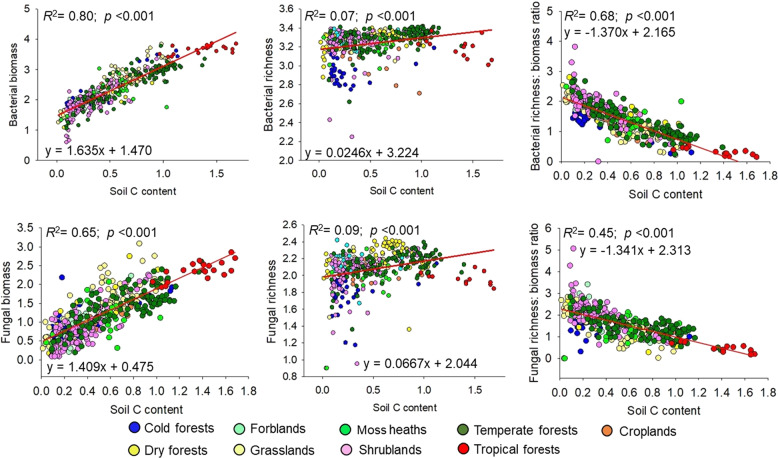
Relationships between soil carbon content (%), microbial biomass (nmol PLFA g^−1^ dry soil), microbial richness, and the richness-to-biomass ratio of bacterial and fungal communities (unitless). All variables are normalized (log_10_ X + 1). *N* = 435 soil samples from 87 globally distributed locations (Fig. S1). Major biomes are based on field vegetation and climatic information from Kottek et al. [81].

We first show that the relationship between soil microbial biomass and diversity follows a unimodal (humped-back) pattern across global biomes (Fig. 1, Table S2). Moreover, the humped-back relationship between biomass and diversity also occurs when removing tropical soils which showed the highest biomass (Fig. S4). We then used SEM to further investigate the environmental factors associated with the relationship between microbial diversity and biomass (Fig. 2). These analyses revealed that soil C content indirectly determines microbial diversity via changes in microbial biomass (Fig. 2; Tables S3 and S4) and it is a fundamental driver of the diversity-to-biomass ratio both for bacterial and fungal communities (Fig. 3; Fig. S5; Tables S5 and S6). Further, soil C content correlated positively with both microbial biomass and microbial diversity, but correlations were much higher for the relationship between soil C content and microbial biomass than for richness (Fig. 4). Indeed, the slopes of the linear relationships between soil C and microbial biomass were higher than those between soil C content and microbial richness (Fig. 4). These results suggest a stronger effect of soil C content over microbial biomass than richness which ultimately determines the negative relationship between soil C content and richness-to-biomass ratios across global biomes. Moreover, we converted our PLFA data into microbial biomass C, using the equation provided by Bailey et al. [56]. As in the case of PLFAs, we found that the slope of the relationship between soil C content and microbial biomass C (0.041) was slightly higher than that previously reported by Fierer et al. [57] (0.013) and the one obtained from the meta-analysis by Xu et al. [58] (0.009) (Fig. S6; Table S7). This discrepancy could originate from the different methods utilized for estimating microbial biomass (microbial biomass C vs. PLFAs), the different number of samples considered in each study, and the fact that the study of Xu et al. [58] is based on a meta-analysis and results can be more difficult to compare with direct estimates. In any case, the greater slope observed in our study could suggest that variations in soil C content as a consequence of changes on land use, deforestation or climate may potentially have more drastic influence on microbial biomass than previously reported. Nevertheless, causational relationships between soil C content and microbial biomass cannot be easily deciphered because soil carbon does not only derive from aboveground, and microbial biomass and microbial metabolites also contribute to stabilization of C in soil by forming associations with soil minerals [59–61].

Moreover, our results indicate that the negative relationship between soil C content and the richness-to-biomass ratio occurs across independent ecosystems, with the unique exception of the fungal community of moss heaths (Figs. S7 and S8). Thus, soils from locations with high C content (e.g., tropical regions) were associated with more microbial biomass and comparatively lower richness (Fig. 1) than soils with lower soil C content (e.g., cold and arid grasslands). Of course, other drivers, such as soil pH were essential drivers of microbial richness across our soils [23, 45, 62] (Fig. S9). However, our SEM approaches provided evidence that soil C content has a greater importance than other variables in the regulation of the richness-to-biomass ratio of bacterial and fungal communities (Figs. 2–3; Fig. S5; Tables S3–S6). Indeed, SEM approach indicates that soil C content indirectly regulates soil microbial richness via a positive association with microbial biomass. Together, these results highlight that soil C content has an important role in shaping the relationship between soil microbial diversity and biomass, although other soil parameters (i.e., pH and texture) may contribute to the observed patterns.

Two main conceptual alternatives derived from plant ecology (Stress Gradient Hypothesis and competitive exclusion) are the most parsimonious mechanisms explaining the reported humped-back associations between soil microbial diversity and biomass across global biomes. The Stress Gradient Hypothesis predicts that positive species interactions such facilitation are more important in stressful environments than in more benign ones where competition should be more common [9, 29, 30]. Thus, in more stressful soil environments, such those located in more arid environments, with relatively poor soil C content (i.e., shrublands, dry and cold forests), facilitation and niche partitioning through specialization support the co-existence of multiple microbial species, and increases in soil C content and microbial biomass were positively associated with soil microbial richness. This type of relationship has previously been described in low C dryland ecosystems [63]. In our study, microbial diversity peaked in grasslands (86% located in cold and arid regions), which generally had intermediate levels of microbial biomass. Further, the biomass of bacteria and fungi was particularly high in tropical forests, but microbial diversity was low when compared with other ecosystems (i.e., cold, temperate and dry forests, grasslands, moss heaths and shrublands) (Fig. 1). This result is in agreement with previous studies reporting relatively low levels of bacterial [64] and fungal [65] richness in tropical forest soils compared with more temperate regions. Our results thus support the notion that, as soil C content increases, microbial biomass and likely the abundance of dominant taxa are promoted, which in turn reduces the diversity of subordinate taxa via competitive exclusion, resulting in an overall reduction in species richness of a given soil [66–68]. Further, theoretical plant ecology provides support for this finding because there is evidence that dominant plant species may suppress the diversity by preventing the establishment of other species [8–10, 69].

These results are integral for us to predict changes in soil biodiversity globally, as C is highly threatened by climate change and land use intensification [70, 71]. Our findings suggest that moderate reductions in soil C content of high C soils could led to reductions in microbial biomass and unexpected increases in microbial diversity. Thus, reductions of soil C content and microbial biomass via deforestation, land clearing and cropping, warming or aridity [28, 71–73] might result in increases in microbial diversity by releasing subordinate taxa. Such an effect has been reported previously at local scales, for example, in response to deforestation in tropical and subtropical forests [74, 75] and in Mediterranean ecosystems [76]. However, our results also suggest that reductions in soil C content to very low levels, for instance as a result of increased aridity caused by climate change [77], could lead to simultaneous reductions in both microbial biomass and diversity, and their attendant ecosystem effects (e.g., in arid ecosystems; [63]). These findings are essential to predict how soil organic C and microbial biomass will influence soil microbial diversity and the potential consequences that such changes can have in ecosystem functionality [32, 33, 78].

**Fig. 5:**
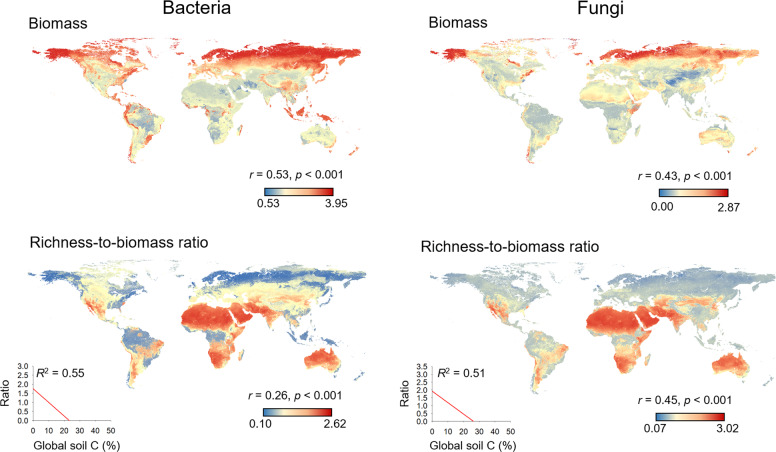
Predicted global distribution of biomass and standardized richness-to-biomass ratio of soil bacterial and fungal communities (unitless). Microbial biomass units are nmol PLFA g^−1^ dry soil. All variables are normalized (log_10_ X + 1). An alternative version of this figure showing qualitative data can be found in Fig S11.

Our results also provide novel evidence that bacterial and fungal richness-to-biomass ratios are strongly negatively correlated with the soil C content across global biomes (Fig. 4; Table S8), and that this pattern occurs also within-biomes (Figs. S7 and S8) with the only exception of fungal communities in moss heaths. Indeed, compared with space, climate, vegetation and other soil properties, soil C content was the environmental attribute showing the strongest significant correlation with microbial richness-to-biomass ratios (Table S8). Our results also show that the standardized richness-to-biomass ratio of bacteria (see “Methods” section) was highly correlated with the ratio of fungi (Fig. S10), indicating that both fungi and bacteria share similar optimal conditions for biomass and diversity. Moreover, using the strong predictive power of our models, we developed global maps of microbial biomass and the standardized richness-biomass ratio using the Cubist algorithm [64]. We found that the distribution of microbial biomass mirrored that of richness-to-biomass ratios for both bacteria and fungi (Fig. 5; Fig. S11). At the global scale, the richness-to-biomass ratios peaked in arid environments which possess very low C content and microbial biomass (Fig. 5). However, the microbial richness-to-biomass ratios were intermediate or low in tropical regions and on boreal ecosystems wherein microbial biomass peaked. Soil C content was also strongly and negatively correlated with the predicted distribution of the richness-to-biomass ratios for bacteria (*r* = −0.93; *p* < 0.001) and fungi (*r* = −0.85; *p* < 0.001) at a global scale.

**Fig. 6:**
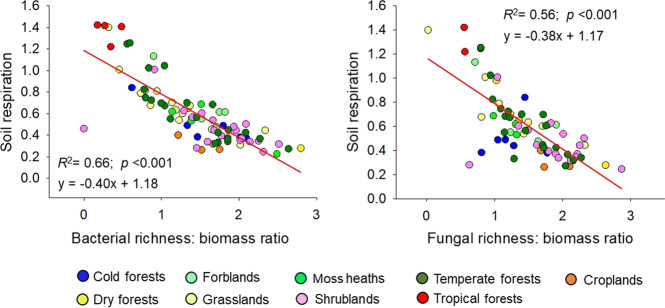
Relationship between soil respiration and the richness-to-biomass ratio (unitless) of soil bacterial and fungal communities. *N* = 86. All variables were normalized (log_10_ X + 1).

Our results also identify novel insights into how soil microbial diversity and biomass might control ecosystem functions. We show that the standardized richness-to-biomass ratio (for both bacterial and fungi) was negatively correlated with soil respiration (Fig. 6), even after controlling for soil C content using partial correlations (bacteria: *r* = −0.26, *p* < 0.001; fungi: *r* = −0.32, *p* < 0.001). Bacterial and fungal biomass were correlated significantly with soil respiration (bacteria: *r* = 0.867, *p* < 0.001; fungi: *r* = 0.872, *p* < 0.001), while only fungal richness correlated significantly with soil respiration (*r* = 0.343, *p* = 0.031) (Fig. S12). Moreover, the slopes of the linear equations were higher for the relationship between microbial biomass and respiration than in the case of richness (Fig. S12). Our results are in agreement with previous experimental and meta-analysis studies suggesting that microbial biomass is a fundamental microbial attribute controlling broad soil processes [15, 79, 80], and suggest that, as soils reduce their biomass compared with diversity, critical broad processes, such as soil respiration are negatively affected. On the contrary, it is plausible to consider that soils that maintain comparatively higher biomass than diversity (lower diversity-to biomass ratios) can be associated to higher respiration rates. Our results suggest that the soil microbial diversity-to-biomass ratio can contribute to explain changes in soil respiration across the globe. Further, these findings imply that any anthropogenic activities that substantially imbalance the microbial richness-to biomass ratio may have potential consequences for ecosystem services supported by the soil, particularly CO_2_ release from soil to atmosphere.

Together, our study highlights the importance of soil C content as the major regulator of the relationship and ratio between soil microbial diversity and biomass across contrasting biomes, and that variations in the microbial richness-to-biomass ratio can have negative consequences for ecosystem functioning. This work provides strong evidence that reductions in the soil microbial richness-to-biomass ratio (via climate change and deforestation) will affect critical soil functions that are associated with the regulation of Earth’s climate.

## Supplementary information

Supporting Information

## Data Availability

Data associated with this study will be publicly available in (https://figshare.com/s/b75f1c08ceca22aa551b) upon manuscript acceptance.
