# Supplementary material for: Soil microbial diversity–biomass relationships are driven by soil carbon content across global biomes
Source: ISME J. 2021 Feb 9;15(7):2081–91. doi: 10.1038/s41396-021-00906-0 (PMC8245509; doi:10.1038/s41396-021-00906-0)
Supplement: Supplementary file 1 — Supporting Information [file 41396_2021_906_MOESM1_ESM.docx]

**Supporting Information**

**Soil microbial diversity-biomass relationships are driven by soil carbon content across global biomes**

Felipe Bastida^1,*^, David J. Eldridge^2^, Carlos García^1^, G. Kenny Png^3,4^, Richard D. Bardgett^3^, Manuel Delgado-Baquerizo^5^.

^1^CEBAS-CSIC. Department of Soil and Water Conservation. Campus Universitario de Espinardo, 30100, Murcia, Spain.

^2^Centre for Ecosystem Studies, School of Biological, Earth and Environmental Sciences, University of New South Wales, Sydney, New South Wales, 2052, Australia.

^3^Department of Earth and Environmental Sciences, Michael Smith Building, The University of Manchester, Oxford Road, Manchester M13 9PT, UK.

^4^Asian School of the Environment, Nanyang Technological University, 50 Nanyang avenue, Singapore 639798.

^5^Departamento de Sistemas Físicos, Químicos y Naturales, Universidad Pablo de Olavide, 41013, Sevilla, Spain.

***Corresponding author:** Felipe Bastida ([fbastida@cebas.csic.es](mailto:fbastida@cebas.csic.es))

This document contains supplemental tables and figures which are cited in the corresponding manuscript, including the global survey map, *a priori* SEM model and several results and statistical analyses.

**
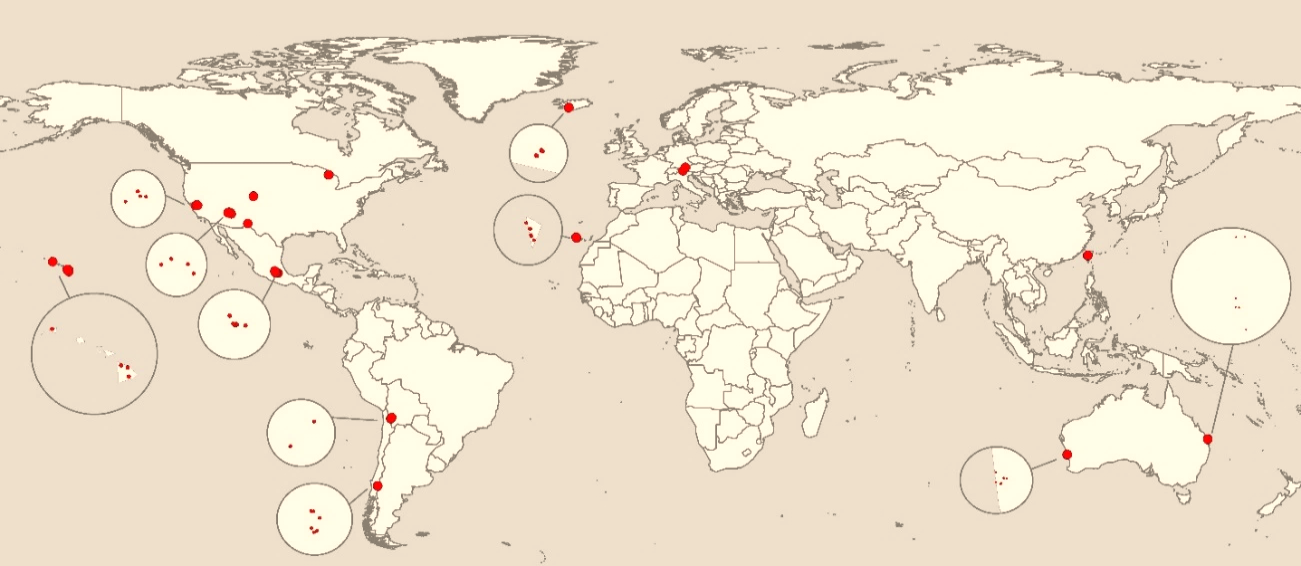
**

**Figure S1.** Location for the 87 globally-distributed sites included in our study.


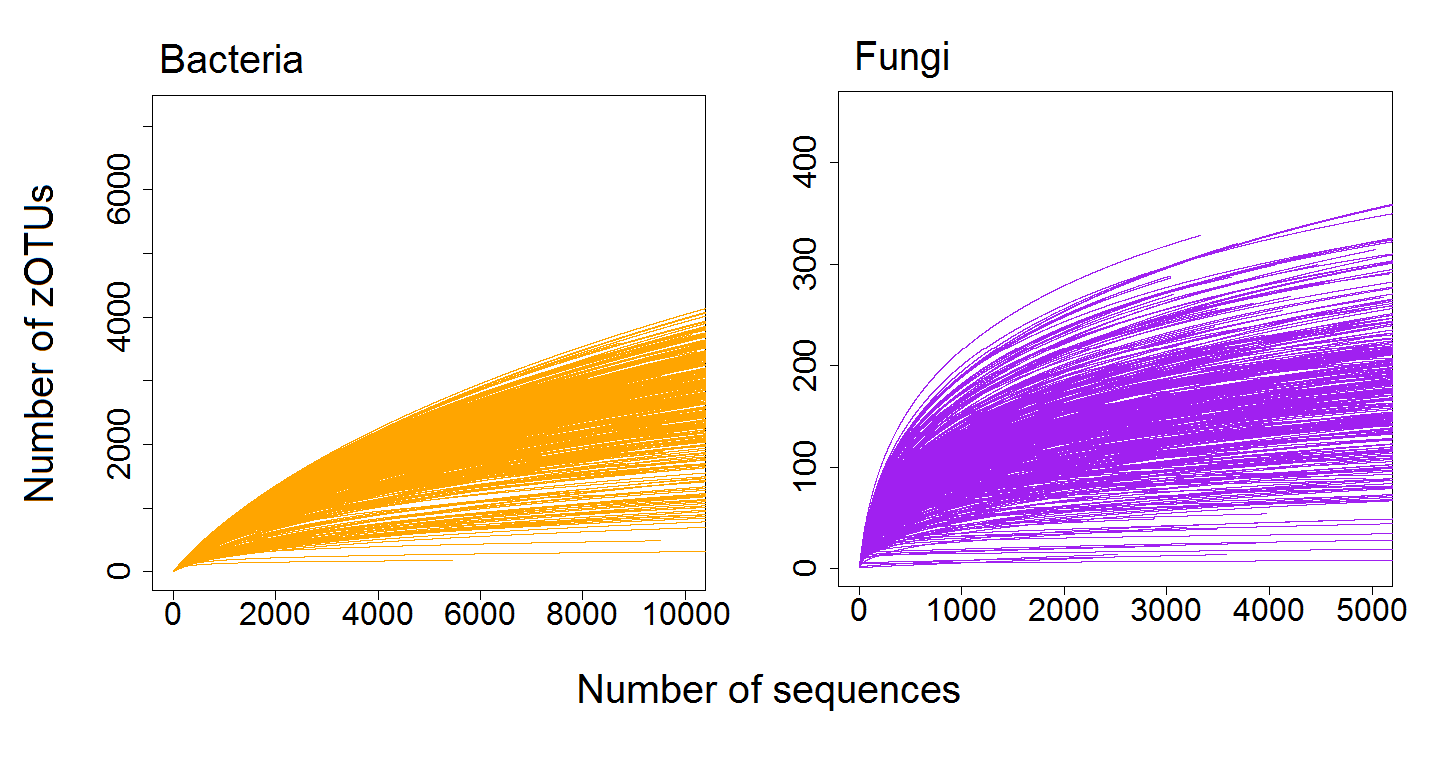


**Figure S2.** Raferaction curves for bacterial and fungal communities in this study.

**
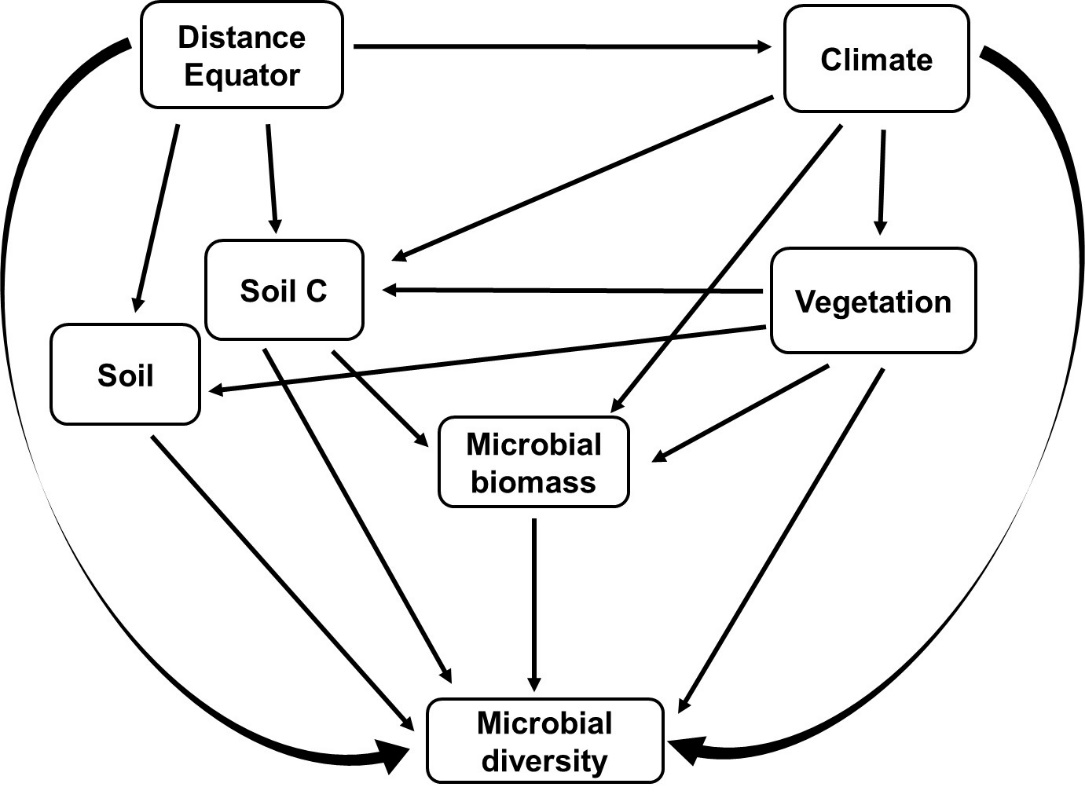
**

**Figure S3.** *A priori* structural equation modeling (SEM) describing the effects of multiple predictors on soil microbial diversity. Climate includes mean annual precipitation (MAP) and mean annual temperature (MAT). Soil includes pH and texture. Vegetation includes plant cover, grassland and forest. See Table S1 for a complete list of variables.


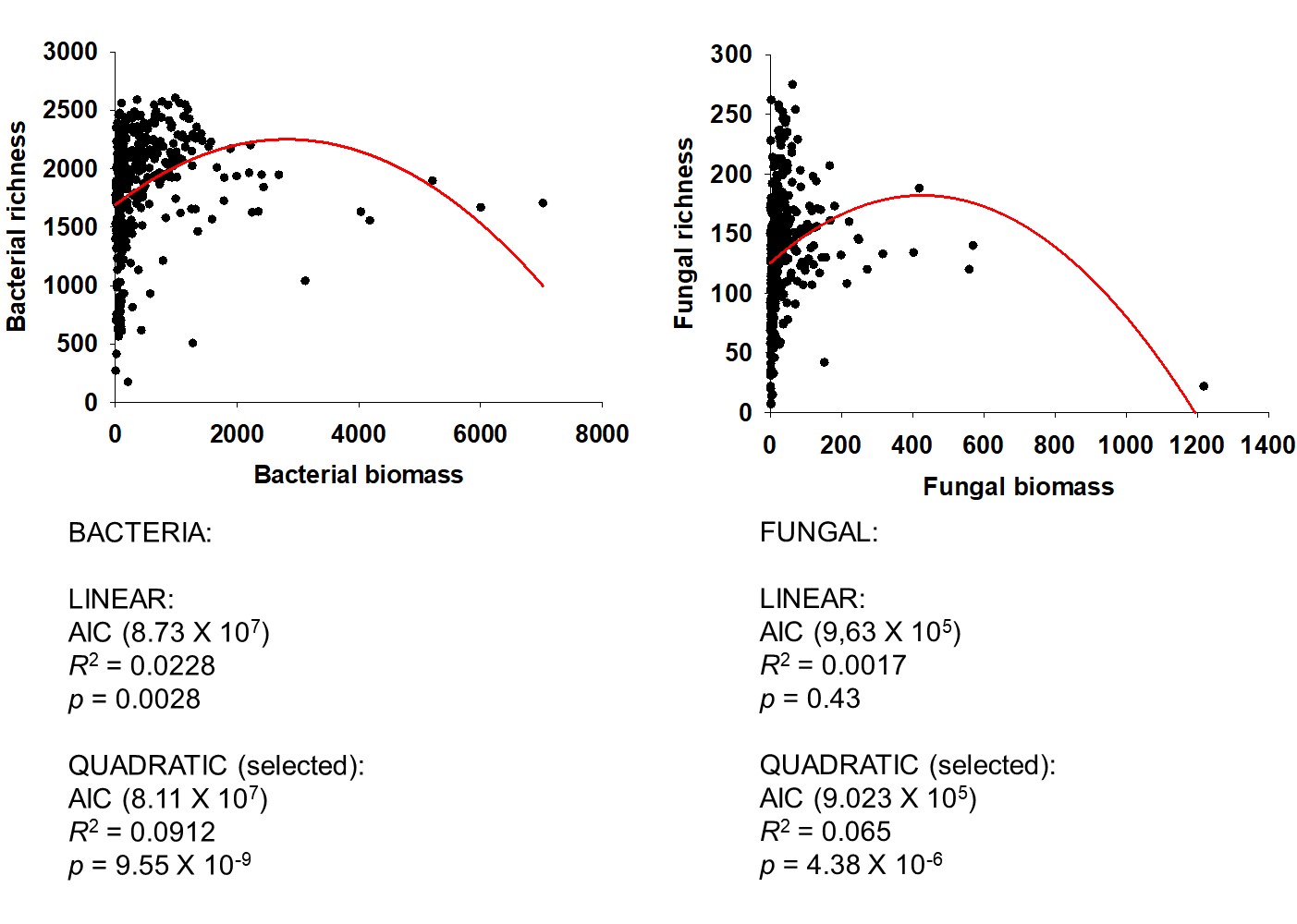


**Figure S4.** Diversity to biomass relationships in the global dataset without accounting with tropical soils.


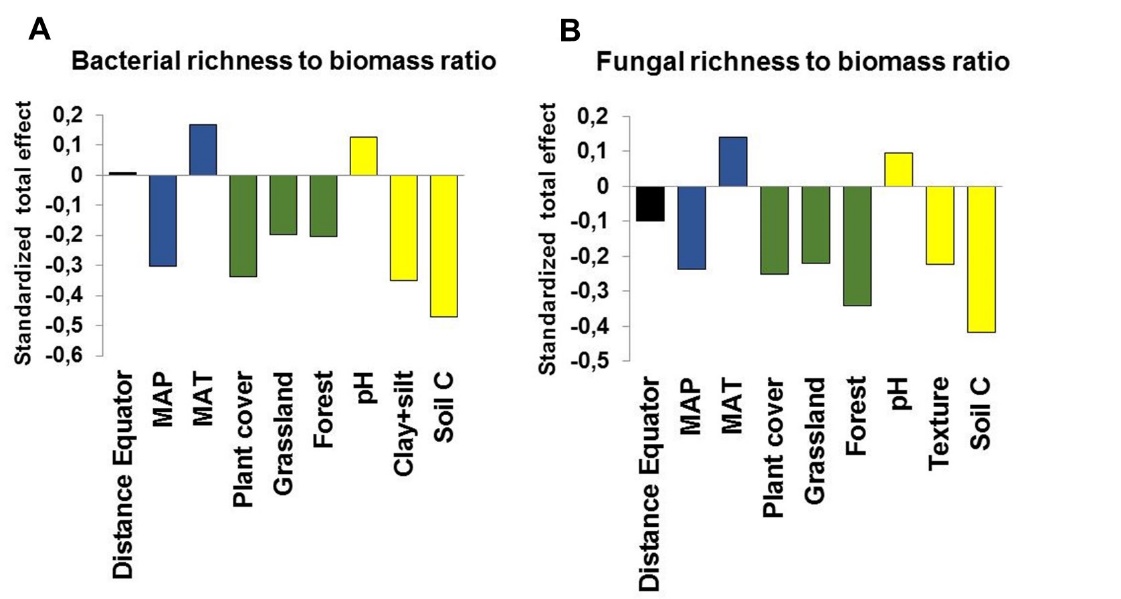


**Figure S5.** Standardized total effects (STE) of different variables in microbial richness to biomass ratio.


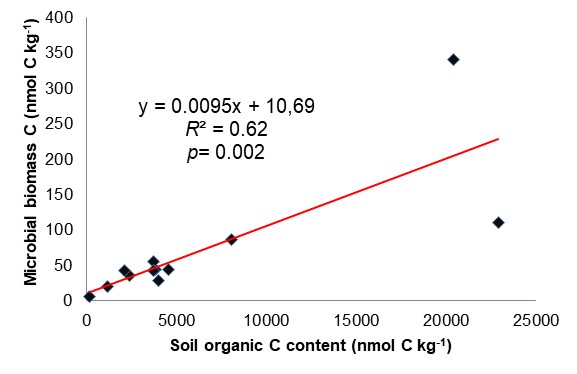


**Figure S6.** Relationship between soil C content and microbial biomass C obtained from the within-biomes averaged values showed by Xu et al. (2013).


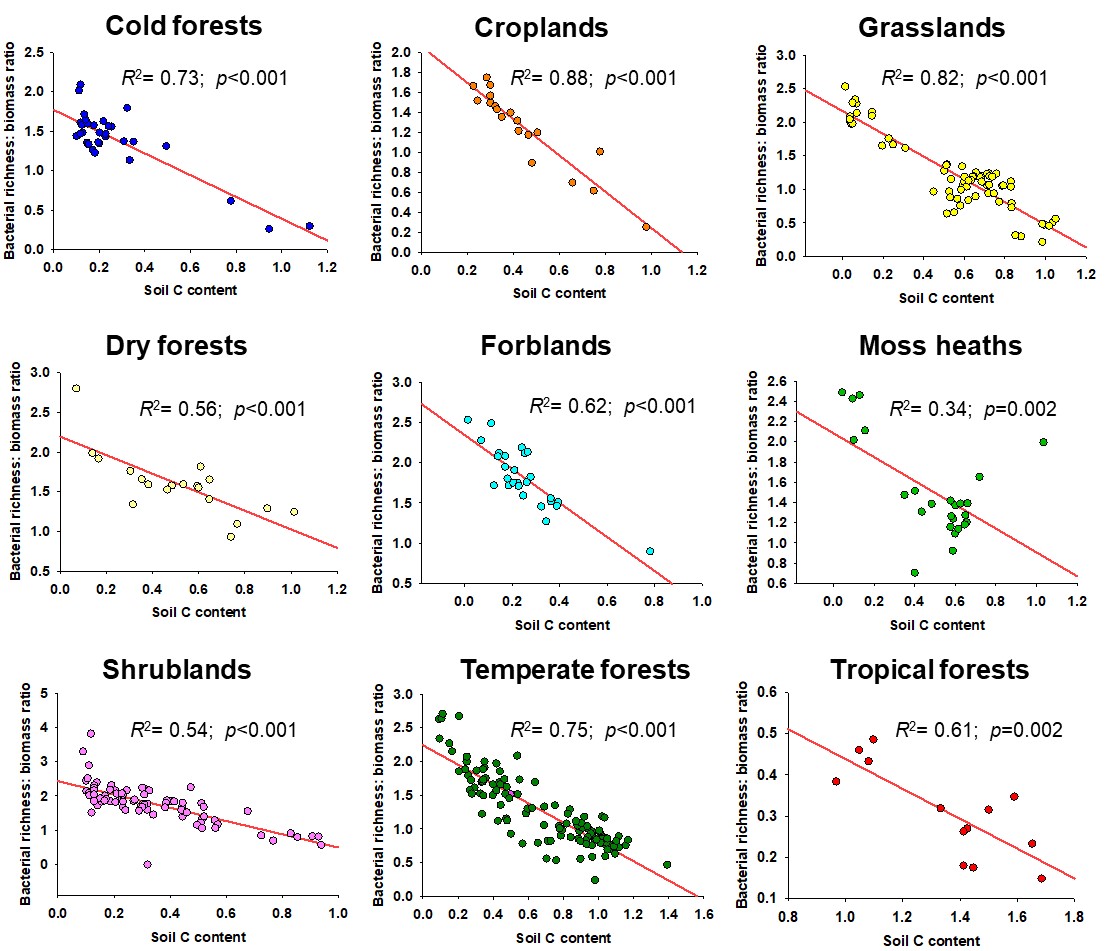


**Figure S7.** Relationships between soil C content and bacterial richness:biomass ratio within biomes. Values in x- and y-axes are transformed (log_10_ X +1).


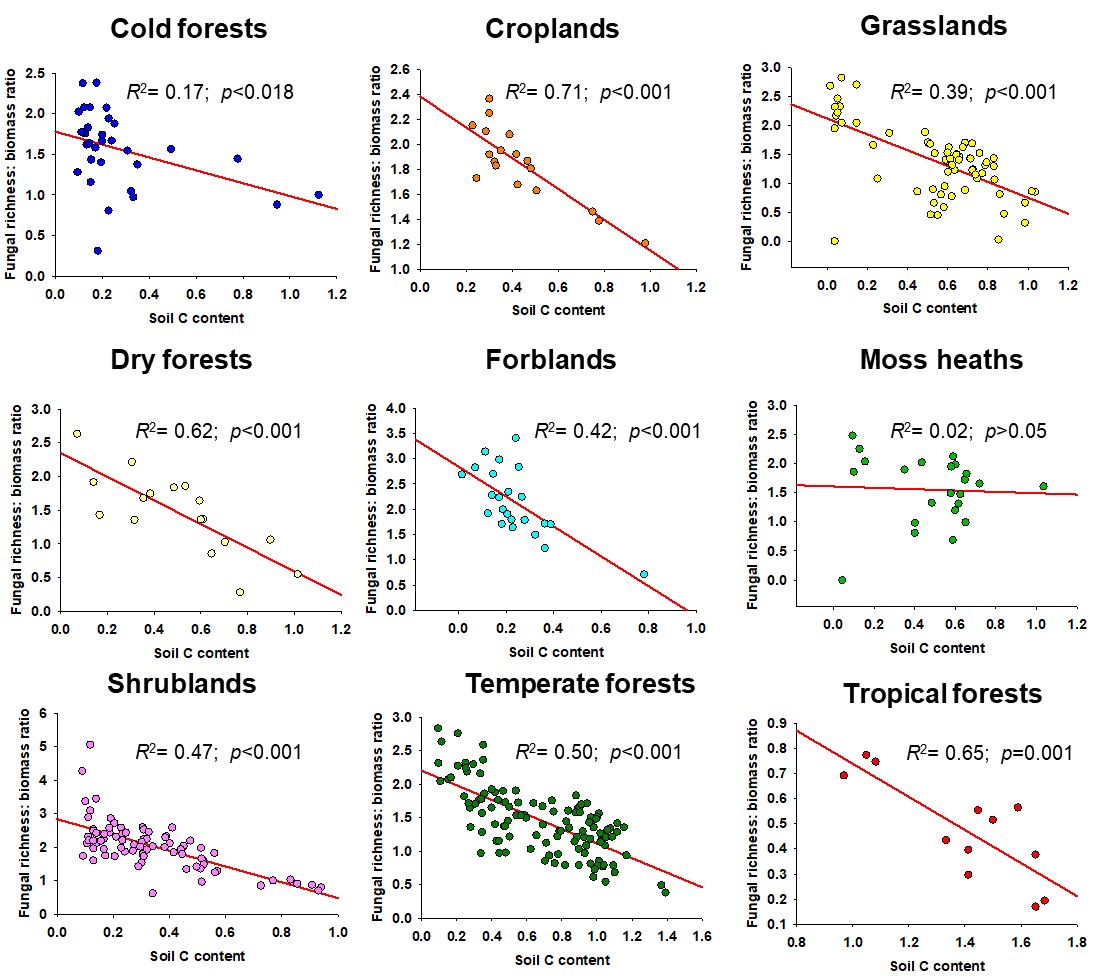


**Figure S8.** Relationships between soil C content and fungal richness:biomass ratio within biomes. Values in x- and y-axes are transformed (log_10_ X +1).

**
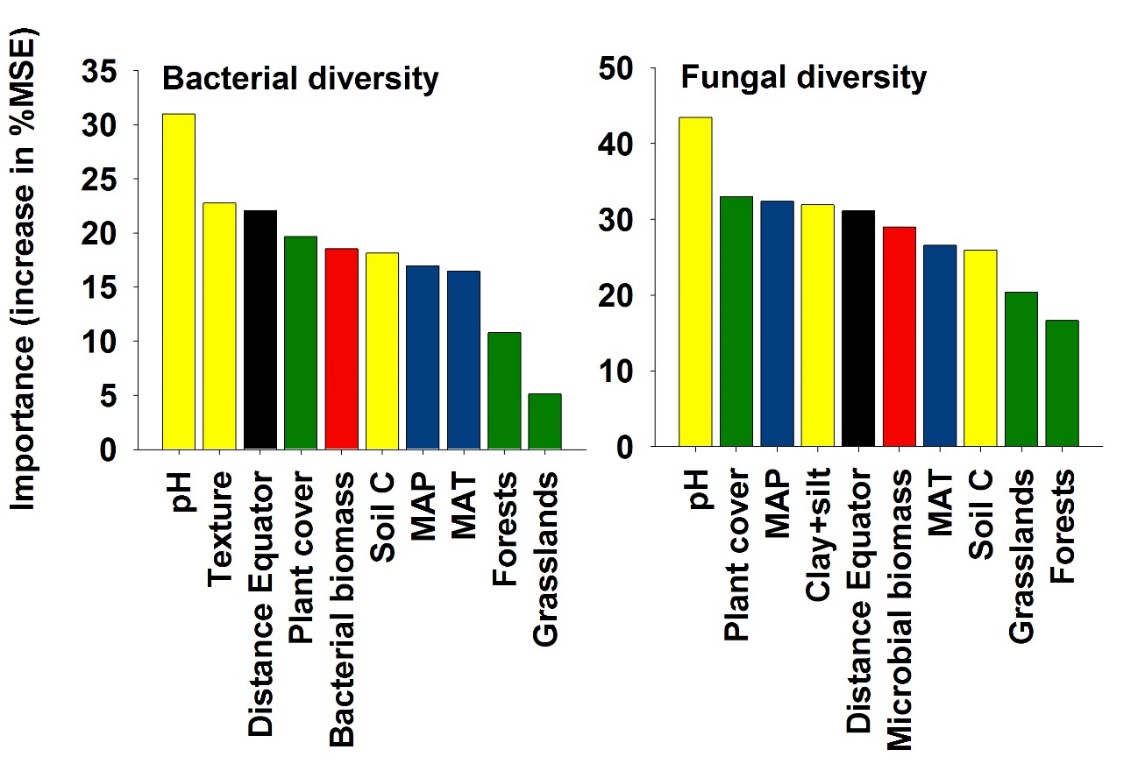
**

**Figure S9.** Predictors of microbial diversity across globally distributed soils, identified using random forest modeling. MSE (Mean Square Error). MAP (Mean annual precipitation), MAT (Mean annual temperature).


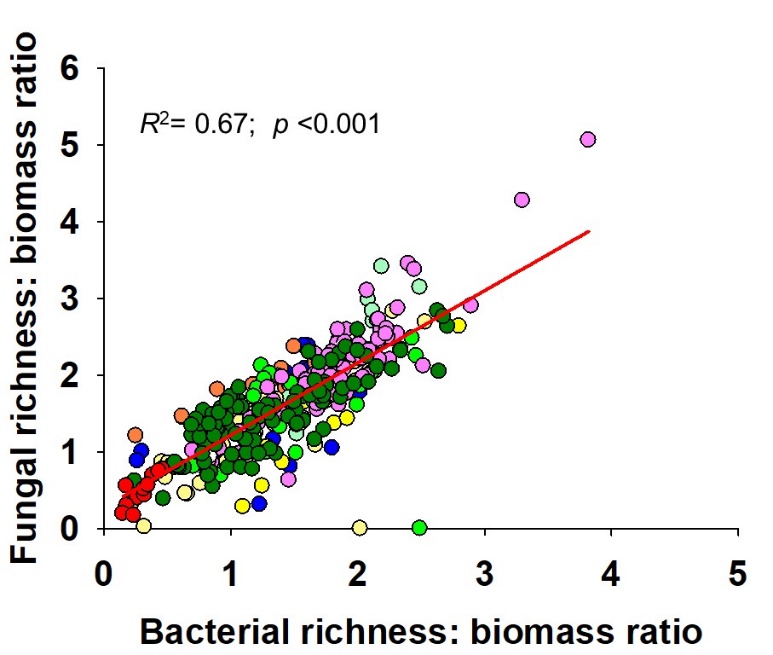


**Figure S10.** Relationship between richness-to-biomass ratio of bacterial and fungal communities across global biomes. Values in x- and y-axes are transformed (log_10_ X +1).


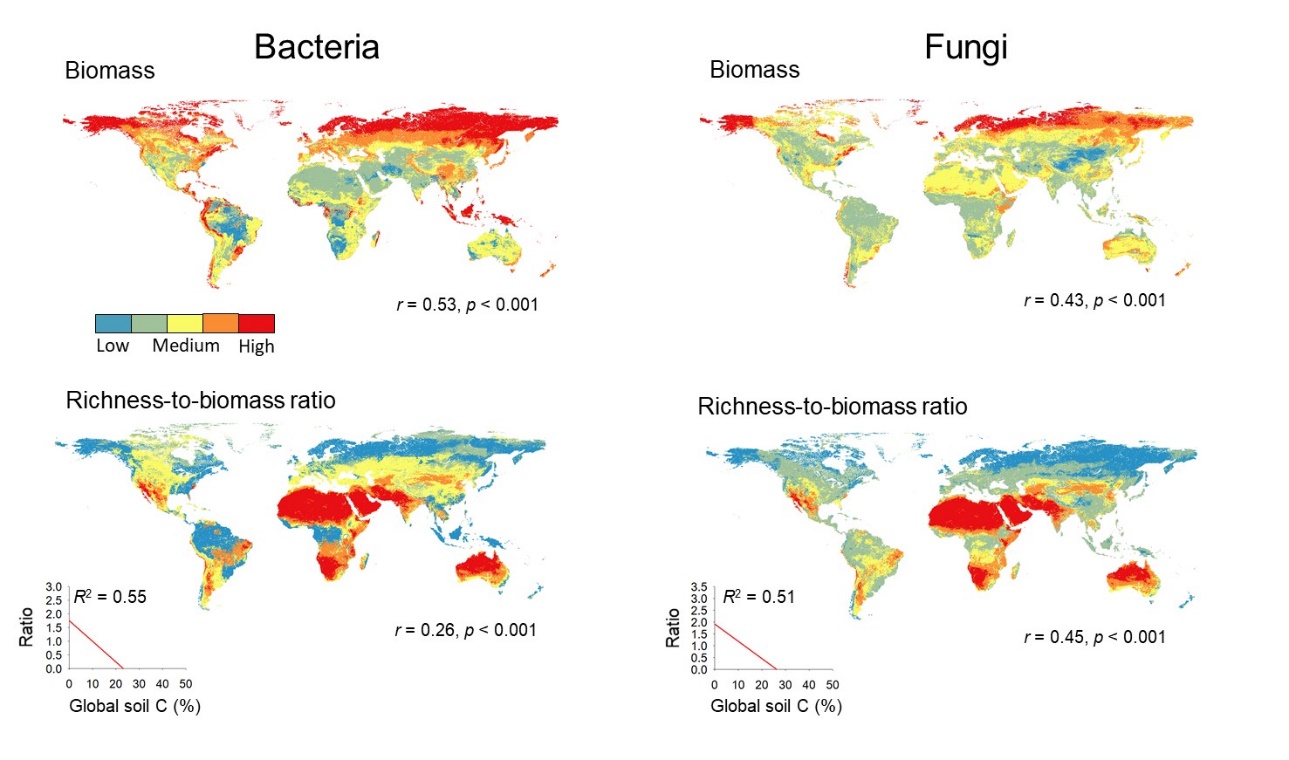


**Figure S11.** Alternative version of Figure 5 showing qualitative data for the predicted global distribution of biomass and standardized richness-to-biomass ratio of soil bacterial and fungal communities.


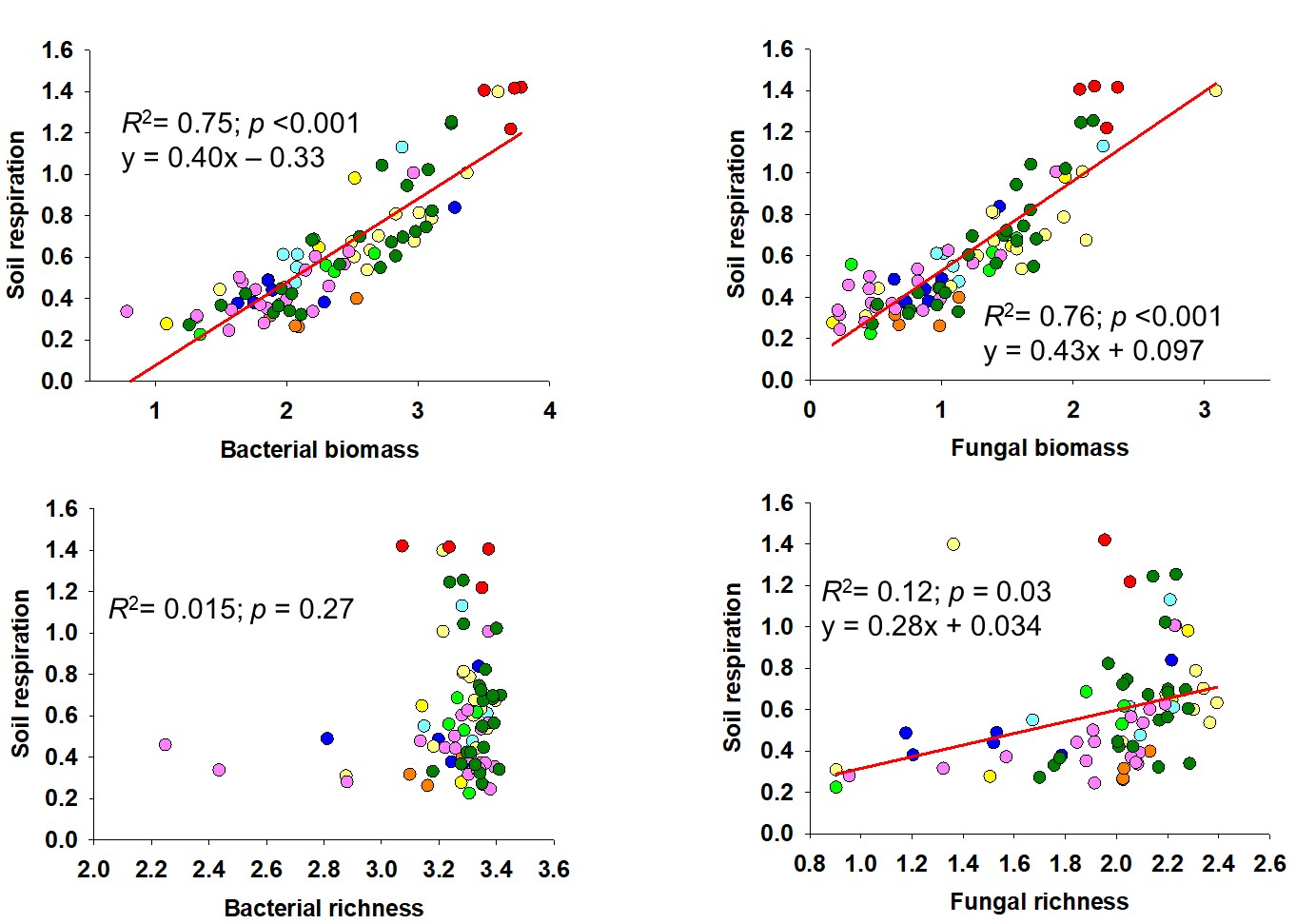


**Figure S12.** Relationship between microbial biomass or richness and soil respiration across global biomes. Values in x- and y-axes are transformed (log_10_ X +1). All tested linear relationships were significant (*p*<0.05) except bacterial richness vs soil respiration.

**Table S1.** Variables included in our SEM.

| Group | Variable | Units |
| --- | --- | --- |
| Space | Distance equator | Decimal degrees |
| Climate | MAT | ºC |
|  | MAP | mm |
| Vegetation | Plant cover | % |
|  | Forest | 0/1 |
|  | Grasslands | 0/1 |
| Soil | Clay+silt | % |
|  | pH | unitless |
|  | Soil carbon | % |
| Microbes | Microbial biomass | Fatty acid (nmol g^-1^ soil) |
|  | Microbial richness | Number of phylotypes (OTUs) |

**Table S2**. Summary for the best models predicting the relationship between microbial biomass and diversity. Models are ranked by significance, AIC and parsimony. AIC measures the relative goodness of fit of a given model; the lower its value, the more likely the model is correct. AIC denotes the difference between the AIC of each model and that of the best model. We considered two alternative models: linear (i.e. the higher the biomass, the higher the diversity) vs. quadratic (there is an optimal point where both biomass and richness peak).

| Variables | Model | *R*^2^ | *p* value | AIC | Selected model |
| --- | --- | --- | --- | --- | --- |
| Bacteria | Linear | <0.001 | 0.486 | 6106.71 |  |
|  | Quadratic | 0.083 | <0.001 | 6074.19 | x |
| Fungi | Linear | <0.001 | 0.629 | 4100.99 |  |
|  | Quadratic | 0.036 | <0.001 | 4089.045 | x |

**Table S3.** Effect sizes between parameters from the structural equation models (SEMs) provided in Figure 2A for bacterial biomass and richness.

| Parameter | | | Direct effect | *p* |
| --- | --- | --- | --- | --- |
| MAT | <--- | DE | -0.605 | 0.001 |
| MAP | <--- | DE | -0.021 | 0.635 |
| Plant_cover | <--- | DE | -0.009 | 0.907 |
| Grassland | <--- | DE | 0.251 | 0.001 |
| Forest | <--- | DE | -0.368 | 0.001 |
| Grassland | <--- | MAT | 0.032 | 0.534 |
| Forest | <--- | MAT | -0.127 | 0.02 |
| Grassland | <--- | MAP | -0.279 | 0.001 |
| Forest | <--- | MAP | 0.227 | 0.001 |
| Plant_cover | <--- | MAP | 0.175 | 0.001 |
| Plant_cover | <--- | MAT | 0.363 | 0.001 |
| Soil_C | <--- | DE | -0.267 | 0.002 |
| Texture | <--- | DE | -0.249 | 0.002 |
| pH | <--- | Plant_cover | -0.237 | 0.001 |
| Texture | <--- | Forest | -0.232 | 0.002 |
| pH | <--- | Forest | -0.36 | 0.001 |
| Texture | <--- | Grassland | 0.133 | 0.009 |
| pH | <--- | Grassland | -0.144 | 0.001 |
| Soil_C | <--- | MAT | -0.159 | 0.002 |
| Soil_C | <--- | MAP | 0.197 | 0.001 |
| Texture | <--- | MAT | 0.02 | 0.678 |
| pH | <--- | MAT | -0.221 | 0.001 |
| Texture | <--- | MAP | 0.187 | 0.017 |
| pH | <--- | MAP | -0.472 | 0.001 |
| Soil_C | <--- | Plant_cover | 0.127 | 0.004 |
| Soil_C | <--- | Forest | 0.215 | 0.002 |
| Soil_C | <--- | Grassland | 0.117 | 0.001 |
| pH | <--- | DE | -0.245 | 0.001 |
| bacterial biomass | <--- | DE | 0.028 | 0.477 |
| bacterial biomass (quadratic) | <--- | DE | -0.098 | 0.075 |
| soil_C (quadratic) | <--- | DE | -0.193 | 0.005 |
| bacterial biomass | <--- | Soil_C | 0.785 | 0.001 |
| bacterial biomass | <--- | Plant_cover | 0.014 | 0.688 |
| bacterial biomass | <--- | Forest | 0.024 | 0.564 |
| bacterial biomass | <--- | Grassland | 0.152 | 0 |
| bacterial biomass | <--- | pH | 0.021 | 0.67 |
| bacterial biomass | <--- | Texture | -0.018 | 0.643 |
| bacterial biomass | <--- | MAP | 0.126 | 0.002 |
| bacterial biomass | <--- | MAT | -0.015 | 0.799 |
| soil pH (quadratic) | <--- | DE | -0.015 | 0.682 |
| Richness_bacteria | <--- | DE | -0.126 | 0.055 |
| Richness_bacteria | <--- | Texture | 0.152 | 0.003 |
| Richness_bacteria | <--- | Plant_cover | 0.211 | 0.001 |
| Richness_bacteria | <--- | Forest | 0.041 | 0.464 |
| Richness_bacteria | <--- | Grassland | 0.075 | 0.088 |
| Richness_bacteria | <--- | MAT | 0.087 | 0.132 |
| Richness_bacteria | <--- | MAP | 0.201 | 0.001 |
| Richness_bacteria | <--- | Biomass (linear+quadratic) | -0.189 | 0.004 |
| Richness_bacteria | <--- | soil C (linear+quadratic) | 0.361 | 0.004 |
| Richness_bacteria | <--- | soil pH (linear+quadratic) | 0.755 | 0.001 |

**Table S4.** Effect sizes between parameters from the structural equation models (SEMs) provided in Figure 2B for fungal biomass and richness.

|  | Parameter |  | Direct effect | *p* |
| --- | --- | --- | --- | --- |
| MAT | <--- | DE | -0.601 | 0.002 |
| MAP | <--- | DE | -0.038 | 0.446 |
| Plant_cover | <--- | DE | 0.004 | 0.947 |
| Grassland | <--- | DE | 0.241 | 0.001 |
| Forest | <--- | DE | -0.386 | 0.001 |
| Soil_C | <--- | DE | -0.274 | 0.003 |
| Texture | <--- | DE | -0.26 | 0.001 |
| pH | <--- | DE | -0.242 | 0.001 |
| Fungal biomass | <--- | DE | 0.212 | 0.003 |
| Richness_fungi | <--- | DE | 0.131 | 0.034 |
| Texture | <--- | Forest | -0.222 | 0.001 |
| pH | <--- | Forest | -0.371 | 0.001 |
| Soil_C | <--- | Forest | 0.197 | 0.001 |
| Fungal biomass | <--- | Forest | 0.175 | 0.045 |
| Richness_fungi | <--- | Forest | 0.031 | 0.621 |
| Richness_fungi | <--- | Fungal biomass (linear and quadratic) | -0.28 | 0.007 |
| Texture | <--- | Grassland | 0.126 | 0.007 |
| pH | <--- | Grassland | -0.139 | 0.001 |
| Soil_C | <--- | Grassland | 0.083 | 0.011 |
| Fungal biomass | <--- | Grassland | 0.196 | 0.003 |
| Richness_fungi | <--- | Grassland | 0.295 | 0.004 |
| Grassland | <--- | MAP | -0.27 | 0.001 |
| Forest | <--- | MAP | 0.233 | 0.001 |
| Plant_cover | <--- | MAP | 0.171 | 0.001 |
| Soil_C | <--- | MAP | 0.197 | 0.001 |
| Texture | <--- | MAP | 0.189 | 0.014 |
| pH | <--- | MAP | -0.485 | 0.001 |
| Fungal biomass | <--- | MAP | -0.087 | 0.041 |
| Richness_fungi | <--- | MAP | 0.033 | 0.55 |
| Grassland | <--- | MAT | 0.005 | 0.925 |
| Forest | <--- | MAT | -0.167 | 0.004 |
| Plant_cover | <--- | MAT | 0.349 | 0.001 |
| Soil_C | <--- | MAT | -0.175 | 0.002 |
| Texture | <--- | MAT | 0.011 | 0.796 |
| pH | <--- | MAT | -0.197 | 0.001 |
| Fungal biomass | <--- | MAT | 0.112 | 0.179 |
| Richness_fungi | <--- | MAT | 0.058 | 0.484 |
| Fungal biomass | <--- | pH | 0.068 | 0.257 |
| Richness_fungi | <--- | pH | 0.404 | 0.002 |
| pH | <--- | Plant_cover | -0.236 | 0.001 |
| Soil_C | <--- | Plant_cover | 0.153 | 0.002 |
| Fungal biomass | <--- | Plant_cover | 0.04 | 0.415 |
| Richness_fungi | <--- | Plant_cover | 0.45 | 0.001 |
| Richness_fungi | <--- | Soil C (linear and quadratic) | 0.339 | 0.022 |
| Fungal biomass | <--- | Soil_C | 0.571 | 0.002 |
| Fungal biomass | <--- | Texture | 0.103 | 0.069 |
| Richness_fungi | <--- | Texture | 0.199 | 0.001 |

**Table S5.** Effect sizes between parameters from the structural equation models (SEMs) provided in Figure 3A for bacterial richness to biomass ratio.

| Parameter | | | Estimate | *p* |
| --- | --- | --- | --- | --- |
| MAT | <--- | DE | -0.605 | 0.001 |
| MAP | <--- | DE | -0.021 | 0.636 |
| Plant_cover | <--- | DE | -0.006 | 0.91 |
| Grassland | <--- | DE | 0.253 | 0.001 |
| Forest | <--- | DE | -0.367 | 0.001 |
| Grassland | <--- | MAT | 0.035 | 0.531 |
| Forest | <--- | MAT | -0.123 | 0.02 |
| Grassland | <--- | MAP | -0.28 | 0.001 |
| Forest | <--- | MAP | 0.225 | 0.001 |
| Plant_cover | <--- | MAP | 0.171 | 0.001 |
| Plant_cover | <--- | MAT | 0.371 | 0.001 |
| Soil_C | <--- | DE | -0.267 | 0.003 |
| Texture | <--- | DE | -0.247 | 0.001 |
| pH | <--- | Plant_cover | -0.233 | 0.002 |
| Texture | <--- | Forest | -0.235 | 0.001 |
| pH | <--- | Forest | -0.36 | 0.001 |
| Texture | <--- | Grassland | 0.131 | 0.01 |
| pH | <--- | Grassland | -0.144 | 0.001 |
| Soil_C | <--- | MAT | -0.159 | 0.002 |
| Soil_C | <--- | MAP | 0.197 | 0.001 |
| Texture | <--- | MAT | 0.026 | 0.581 |
| pH | <--- | MAT | -0.229 | 0.001 |
| Texture | <--- | MAP | 0.185 | 0.006 |
| pH | <--- | MAP | -0.473 | 0.001 |
| Soil_C | <--- | Plant_cover | 0.128 | 0.002 |
| Soil_C | <--- | Forest | 0.215 | 0.001 |
| Soil_C | <--- | Grassland | 0.117 | 0.002 |
| pH | <--- | DE | -0.248 | 0.001 |
| Richness to biomass^1^ | <--- | DE | -0.106 | 0.027 |
| Richness to biomass | <--- | Soil_C | -0.472 | 0.003 |
| Richness to biomass | <--- | Plant_cover | -0.249 | 0.001 |
| Richness to biomass | <--- | Forest | -0.141 | 0.002 |
| Richness to biomass | <--- | Grassland | -0.08 | 0.026 |
| Richness to biomass | <--- | pH | 0.127 | 0.004 |
| Richness to biomass | <--- | Texture | -0.351 | 0.001 |
| Richness to biomass | <--- | MAP | -0.035 | 0.469 |
| Richness to biomass | <--- | MAT | 0.238 | 0.001 |

^1^normalized (log_10_ X +1)

**Table S6.** Effect sizes between parameters from the structural equation models (SEMs) provided in Figure 3B for bacterial richness to biomass ratio.

| Parameter | | | Estimate | *p* |
| --- | --- | --- | --- | --- |
| MAT | <--- | DE | -0.601 | 0.001 |
| MAP | <--- | DE | -0.038 | 0.444 |
| Plant_cover | <--- | DE | 0.008 | 0.926 |
| Grassland | <--- | DE | 0.242 | 0.001 |
| Forest | <--- | DE | -0.384 | 0.001 |
| Grassland | <--- | MAT | 0.008 | 0.875 |
| Forest | <--- | MAT | -0.163 | 0.001 |
| Grassland | <--- | MAP | -0.272 | 0.001 |
| Forest | <--- | MAP | 0.231 | 0.001 |
| Plant_cover | <--- | MAP | 0.167 | 0.001 |
| Plant_cover | <--- | MAT | 0.358 | 0.001 |
| Soil_C | <--- | DE | -0.274 | 0.002 |
| Texture | <--- | DE | -0.258 | 0.001 |
| pH | <--- | Plant_cover | -0.231 | 0.001 |
| Texture | <--- | Forest | -0.225 | 0.001 |
| pH | <--- | Forest | -0.371 | 0.001 |
| Texture | <--- | Grassland | 0.124 | 0.016 |
| pH | <--- | Grassland | -0.139 | 0.001 |
| Soil_C | <--- | MAT | -0.176 | 0.002 |
| Soil_C | <--- | MAP | 0.197 | 0.001 |
| Texture | <--- | MAT | 0.017 | 0.721 |
| pH | <--- | MAT | -0.206 | 0.001 |
| Texture | <--- | MAP | 0.187 | 0.007 |
| pH | <--- | MAP | -0.486 | 0.001 |
| Soil_C | <--- | Plant_cover | 0.154 | 0.002 |
| Soil_C | <--- | Forest | 0.197 | 0.001 |
| Soil_C | <--- | Grassland | 0.082 | 0.008 |
| pH | <--- | DE | -0.245 | 0.001 |
| Richness to biomass^1^ | <--- | DE | -0.249 | 0.001 |
| Richness to biomass | <--- | Soil_C | -0.419 | 0.002 |
| Richness to biomass | <--- | Plant_cover | -0.165 | 0.001 |
| Richness to biomass | <--- | Forest | -0.273 | 0.001 |
| Richness to biomass | <--- | Grassland | -0.144 | 0.002 |
| Richness to biomass | <--- | pH | 0.096 | 0.11 |
| Richness to biomass | <--- | Texture | -0.222 | 0.001 |
| Richness to biomass | <--- | MAP | -0.004 | 0.903 |
| Richness to biomass | <--- | MAT | 0.127 | 0.043 |

^1^normalized (log_10_ X +1)

**Table S7.** Relationship between soil carbon (C) content and soil microbial biomass C in different studies.

| Study | *R*^2^ | *p* value | Equation |
| --- | --- | --- | --- |
| Xu et al. 2013^1^ | 0.62 | 0.0022 | Microbial biomass C = 0.0095 x Soil C + 10.69 |
| Fierer et al. 2009^1^ | 0.91 | 0.001 | Microbial biomass C = 0.013 x Soil C - 42 |
| Our study | 0.66 | < 0.001 | Microbial biomass C = 0.0408 x Soil C + 6.84 |

^1^Data obtained from the within-biomes averaged values showed by Xu et al. (2013) and Fierer et al. 2009. Units: In Xu et al 2013 and our study (microbial biomass C in nmol C kg^-1^ soil; soil C content in nmol C kg^-1^ soil). Fierer et al. 2009 (microbial biomass C in g C m^-2^ soil; soil C content in g C m^-2^ soil).

**Table S8.** Correlations (Spearman) between microbial diversity-to-biomass ratios and state environmental factors. Only correlations with *p* < 0.05 are presented. N = 435.

| Group of variables | Variables | Bacteria | Fungi |
| --- | --- | --- | --- |
| Space | Distance from equator |  |  |
| Climate | MAT |  |  |
|  | MAP | -0.242 | -0.190 |
| Vegetation | Plant cover | -0.408 | -0.341 |
|  | Forest | -0.278 | -0.324 |
|  | Grassland | -0.163 | -0.165 |
| Soil | Clay+silt | -0.502 | -0.313 |
|  | pH | 0.466 | 0.365 |
|  | Soil C | -0.852 | -0.720 |
